# Inflammation of the Lungs

**Published:** 1871-01

**Authors:** 


### INFLAMMATION OF THE LUNGS.

Some of the greatest and best men and women in the land lose their lives every winter from this fearful disease, known professionally as "pneumonia," and in the country as "lung-fever," the operation of which is to fill the blood-vessels of the lungs so full of blood that they distend, and in a sense grow together, becoming a compact mass, almost as solid as flesh, often putting the strongest and most robust men in their graves within a week, often in four days; if death is escaped, there is a painfully slow recovery, now and then in a month or two; in many cases it is years before the health is entirely restored, and in not a few the whole subsequent life-time is made a care, an anxiety, and a misery.

The cause of pneumonia is the application of cold in some form or other to the body, and "settling" on the lungs. Remaining still in a chilly condition an hour or two, getting chilled during sleep, going from a warm room, especially after having been there for several hours, into a cold, raw, damp, out-door air, especially if the wind is blowing against the person,—these are the most frequent causes of the dangerous disease. Tens of thousands of cases could be prevented every year by observing two simple precautions while out of doors. First, keep the mouth resolutely shut on going out, so as to send the air to the lungs by the circuit of the head, thus warming it before it gets there. Second, keep in motion brisk enough to keep off a feeling of chilliness.

